# Emerging role of extracellular vesicles in kidney diseases

**DOI:** 10.3389/fphar.2022.985030

**Published:** 2022-09-12

**Authors:** Huiling Xiang, Chun Zhang, Jing Xiong

**Affiliations:** Department of Nephrology, Union Hospital, Tongji Medical College, Huazhong University of Science and Technology, Wuhan, China

**Keywords:** extracellular vesicles, exosomes, kidney diseases, urinary extracellular vesicles, chronic kidney disease

## Abstract

Many types of renal disease eventually progress to end-stage renal disease, which can only be maintained by renal replacement therapy. Therefore, kidney diseases now contribute significantly to the health care burden in many countries. Many new advances and strategies have been found in the research involving kidney diseases; however, there is still no efficient treatment. Extracellular vesicles (EVs) are cell-derived membrane structures, which contains proteins, lipids, and nucleic acids. After internalization by downstream cells, these components can still maintain functional activity and regulate the phenotype of downstream cells. EVs drive the information exchange between cells and tissues. Majority of the cells can produce EVs; however, its production, contents, and transportation may be affected by various factors. EVs have been proved to play an important role in the occurrence, development, and treatment of renal diseases. However, the mechanism and potential applications of EVs in kidney diseases remain unclear. This review summarizes the latest research of EVs in renal diseases, and provides new therapeutic targets and strategies for renal diseases.

## 1 Introduction

The incidence of kidney disease is increasing and has become a medical burden for many countries (Lozano et al., 2012). Many types of kidney diseases, such as acute kidney injury (AKI), nephrotic syndrome, and diabetic nephropathy, eventually progress to end-stage kidney disease accompanied with many complications (Chawla and Kimmel, 2012). At present, there is no effective treatment for end-stage renal disease, and the main treatment is based on renal replacement therapy.

Extracellular vesicles (EVs) are cell-derived, membrane-bound structures loaded with contents including proteins, lipids, and nucleic acids (Skog et al., 2008). Notably, EVs used to be considered as metabolic waste produced by cells (Cocucci et al., 2009). In 1967, Wolf proposed that ‘the type of platelet dust’ is the basis for activation during platelet storage and confirmed that EVs might be functional (Wolf, 1967). The bioactive components retain the characteristics of prosecretory cells and travel between cells or tissues. After being endocytosed by downstream cells, EVs function as secretory cells and affect the performance of downstream cells (Kalluri and LeBleu, 2020). Many cells, including dendritic cells, mast cells, T cells, B cells, and epithelial cells can produce EVs (Valadi et al., 2007). To date, no cell has been identified that is unable to secrete EVs, indicating that the secretion of EVs by living cells is a universal phenomenon, and EVs-mediated information transmission may be a common feature of communication between cells. EVs from different cells vary in their contents and functions.

Recently, EVs have been found to play an important role in information exchange between cells (Maas et al., 2017). EVs produced out of the cell can be either paracrine to nearby cells or be internalized by other cells at distance. EVs are usually internalized by host cells due to their unique membrane structure and possible receptor-ligand binding mode, thereby affecting the phenotype of host cells (Mulcahy et al., 2014). Recently, this feature of EVs has been explored for their use as a carrier for certain poorly specific drugs, such as nanoparticle, during targeted therapy (Kooijmans et al., 2016; Piffoux et al., 2018). Moreover, exosomes secreted into tissue or body fluids (such as urine) have become a potential early diagnostic target for diseases (Merchant et al., 2017). For example, Wilm’s tumor-1 (WT-1) in urine exosomes can be used as a diagnostic indicator for diabetes (Kalani et al., 2013).

**FIGURE 1:**
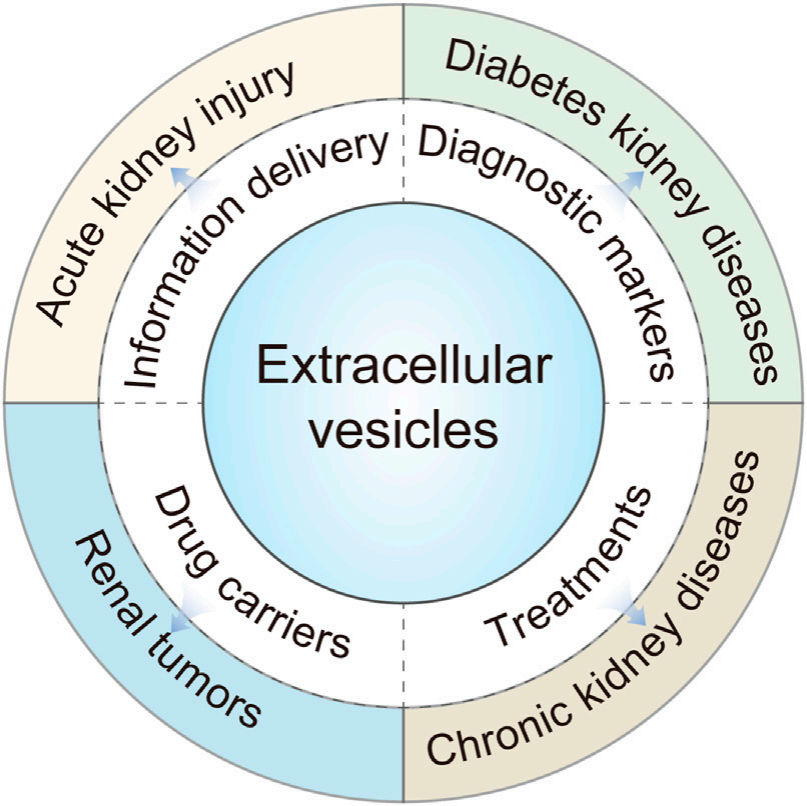
Extracellular vesicles (EVs) play an important role in different types of kidney diseases.

In this review, we emphasize on the updated progress of EVs in kidney disease, focusing on the progress in the research of urinary EVs, the research and application of EVs derived from stem cells in the kidney, and the prospects of diagnosis and treatment of EVs in kidney disease (Erdbrügger and Le, 2016; Aghajani Nargesi et al., 2017; Merchant et al., 2017) (Figure 1).

## 2 The generation, characteristics and isolation secmethods of extracellular vesicles

**FIGURE 2:**
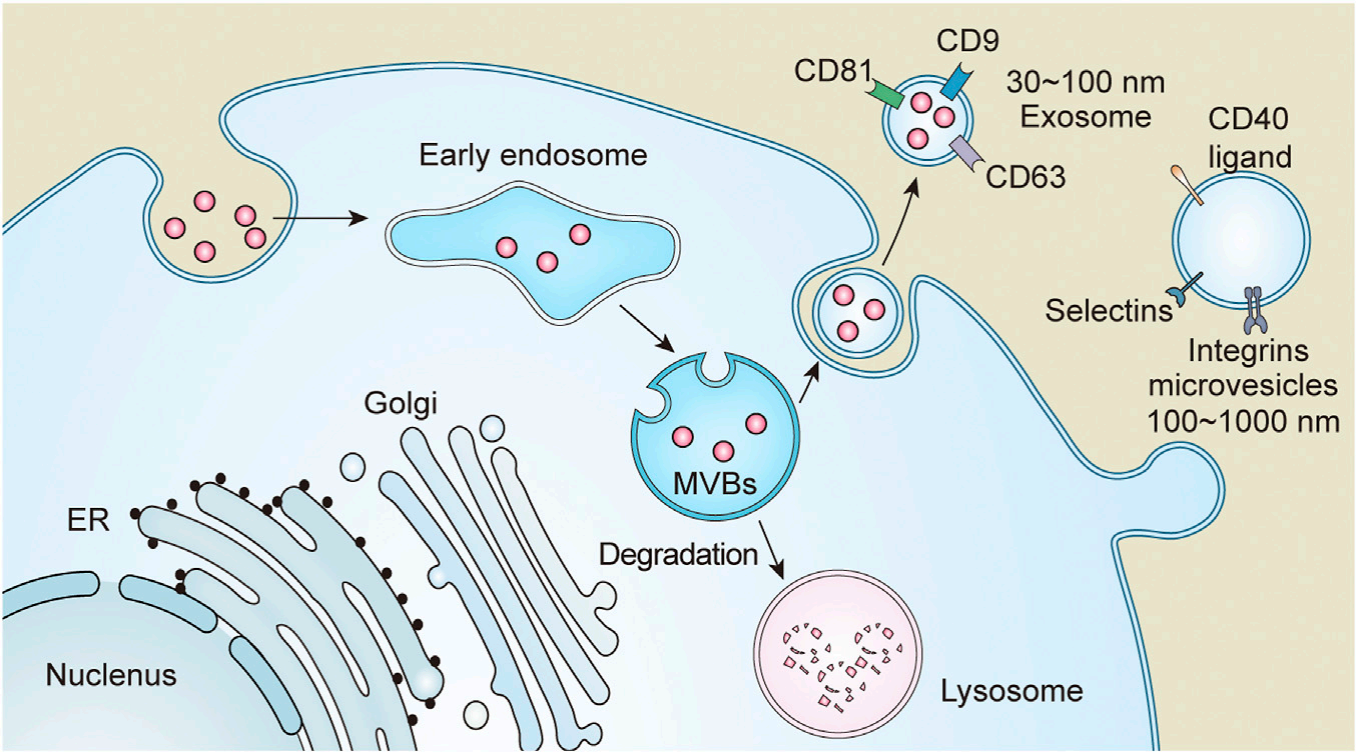
Extracellular vesicles (EVs) are divided into three categories based on size and function, which include exosomes, microvesicles and apoptotic bodies. Endosomes formed after endocytosis, and then become multivesicular bodies (MVBs) through a series of processes. MVBs are partly transported to lysosomes for degradation and partly fused with the plasma membrane or secreted to the outside of the cell to form exosomes due to its special sorting mechanism. Microvesicles are directly detached from the plasma membrane.

Based on their production, size, and function, EVs are currently divided into the following three categories: exosomes, microvesicles, and apoptotic bodies (van der Pol et al., 2012). Active cells generally possess endocytic activity to form endosomes, which are converted into multivesicular bodies (MVBs) through a series of processes. During the maturation of MVBs, a fraction of them degrades, while the remaining fuse with the plasma membrane to release their contents called exosomes (30–100 nm in diameter). Exosomes contain molecules such as heat shock proteins, transmembrane four superfamily (cluster of differentiation (CD)9, CD63, CD81), lipids, and RNAs, etc., (Ohno et al., 2013). Components of the endosomal sorting complex required for transportation are involve in the sorting of MVBs(Colombo et al., 2013). Therefore, interference with endosomal sorting complex required for transportation affects the production of exosomes. Microvesicles, also called exfoliated vesicles, having a volume of 100–1000 nm primarily originate from the cytoplasm. Microvesicles directly shed from the plasma membrane and contain molecules such as phosphatidylserine, integrin, selectin, and CD40 ligand (Lv et al., 2019a). Apoptotic bodies are mainly produced from apoptotic cells, having diameters more than 1000 nm (Caruso and Poon, 2018). The classification and nomenclature of EVs are still controversial, primarily due to limitation of purification methods. Perhaps the nomenclature might evolve with further research. In this review, we have referred to the 2018 EVs guidelines for nomenclature (Théry et al., 2018). All the ongoing research focuses on exosomes and microvesicles. Therefore, this review emphasizes on exosomes and microvesicles in kidney diseases (Figure 2).

There are several extraction methods for EVs, based on their size and biological activity. These include differential ultracentrifugation, density gradient centrifugation, filtration, precipitation, and immunoseparation, and some extraction kits based on these basic principles (Lobb et al., 2015; Vergauwen et al., 2017). Among these, the most commonly used method is ultracentrifugation, which first separates cell debris and other impurities at different speeds and eventually extracts EVs under high-speed conditions. For instance, microvesicles and exosomes can be separated at approximately 10,000 ×g and 100,000 ×g, respectively (Théry et al., 2006). Ultracentrifugation is the most common and accepted method for EVs extraction. High purity exosomes can be extracted by ultracentrifugation, but the conditions of centrifugation are related to the viscosity of the sample (Xu et al., 2016). Samples with higher viscosity, such as serum, are usually centrifuged at longer speeds and times (Livshits et al., 2015). Density gradient centrifugation is usually combined with centrifugation to improve the purity of exosomes. The common carrier is sucrose. Density gradient centrifugation can separate particles with different densities and is suitable for extracting exosomes with low content. However, it is very strict to control the centrifugation time, otherwise it is easy to appear impurity particles. The biggest problem of exosome extraction by filtration method is that a large number of exosomes are lost in the filtration process. Immunoseparation method uses antibody-coated technology to extract and isolate exosomes, but it is not suitable for large sample separation (Li et al., 2017).

All the current extraction methods have specific sample requirements, thus, limiting the application of EVs for treatment or diagnosis. The identification methods for EVs mainly focus on following three aspects: particle size, morphology, and purity (Koliha et al., 2016). Nanoparticle tracking analysis can detect the size and purity of EVs. An electron microscope can determine the morphology as well as also particle size of EVs. Western blotting can identify exosome markers, such as CD63, CD9, and CD81. However, a single method cannot confirm the identity of EVs, and a combination of nanoparticle tracking analysis, electron microscopy, and western blotting is requires (Franzin et al., 2022).

## 3 EVs and kidney diseases

Although majority of the research on EVs in the kidney focuses on urinary EVs and stem cell-derived EVs, some recent studies have focused on the EVs of renal intrinsic cells and perirenal cells (Dominguez et al., 2017). EVs secreted by these cells are also involved in communication and the development of kidney diseases (Camus et al., 2012). Although the existing research provides basic knowledge, further in-depth research regarding the possible mechanism are urgently needed. Here, EVs mainly act as a messenger of information exchange (Gildea et al., 2014).

### 3.1 The role of extracellular vesicles in acute kidney injury

In AKI, the current research focuses on the role of EVs produced by renal tubular epithelial cells and those derived from vascular endothelial cells (Livingston and Wei, 2016; Dominguez et al., 2018). *In vitro* studies, there have revealed that direct administration of the epithelial-derived exosome activating transcription factor 3 RNA can alleviate I/R renal injury by directly inhibit the expression of monocyte chemotactic protein-1 (Chen et al., 2014). Interestingly, this study demonstrated that EVs derived from renal tubular epithelial cells function in an autocrine manner. However, under nonoptimal conditions, EVs produced by renal tubular epithelial cells seem to play a negative role. For example, exosomes from albumin-stimulated renal tubular epithelial cells could activate macrophages and induce renal tubular interstitial inflammation through CCL2 mRNA (Lv et al., 2018). Damaged epithelial cells produce exosomes containing transforming growth factor beta 1, which activates fibrosis (Borges et al., 2013). Hypoxic renal tubular epithelial cells can release miRNA-23a-rich exosomes which activate macrophages triggered their reprogramming into a pro-inflammatory state via suppression of the ubiquitin editor A20, causing tubular interstitial inflammation (Li et al., 2019). The microparticles produced by the activated vascular endothelium can increase the expression of hypoxia-inducible factors in human proximal tubular epithelial cells; however, the beneficial or harmful effects of this activation may depend on different pathological conditions and need further investigation (Fernandez-Martínez et al., 2014). Moreover, platelet-derived microvesicles secrete miRNA-191 to induce apoptosis of renal tubular epithelial cells in I/R AKI (Wu et al., 2019).

### 3.2 The role of extracellular vesicles in chronic kidney disease

In chronic kidney disease (CKD), EVs from damaged tubular epithelial cells promote fibrosis (Liu et al., 2019). Interestingly, tubular cell-derived exosomes have also been found to promote cystic formation in polycystic kidneys (Ding et al., 2021). These exosomes may promote fibrosis by promoting fibroblast activation and recruiting macrophages. An increase in the levels of circulating particle D factor may be related to complement activation, while an increase in circulating platelet EVs may be related to coagulation (Trappenburg et al., 2012; Jalal et al., 2018). In patients with CKD undergoing hemodialysis, hemofiltration is better than ordinary dialysis. This could be because hemofiltration can significantly reduce the numbers of miRNA 223 in circulation and reduce vascular calcification (Abbasian et al., 2018). In addition to the inherent kidney cells that can activate inflammation, the EVs produced by inflammatory cells under certain conditions also play a role. For example, the exosomes produced by macrophages stimulated by calcium oxalate monohydrate have a pro-inflammatory effect and enhance renal cell interleukin (IL)-8 production and the migration of neutrophils (Singhto et al., 2018; Singhto and Thongboonkerd, 2018). *In vitro*, the EVs from TNF-α-stimulated monocytes can induce inflammation and proteinuria in human podocytes (Eyre et al., 2011).

### 3.3 The role of extracellular vesicles in diabetes nephropathy

In diabetes nephropathy, the difference in circulating EVs, particularly miRNA, is indicative of diabetes (La Marca and Fierabracci, 2017). Under high glucose conditions, mesangial cells undergo apoptosis, which may be caused by miR-15b-5p induction, and macrophage exosomes activate the glomerular mesangial cell via the transforming growth factor beta 1/Smad3 pathway (Zhu et al., 2019; Tsai et al., 2020). In addition, berberine can reduce the release of exosomes from mesangial cell and mesangial cell damage through the transforming growth factor beta 1-PI3K/AKT pathway in high glucose condition (Wang et al., 2018). The exosomes of glomerular endothelial cells can also trigger epithelial-mesenchymal transition and podocyte dysfunction under high glucose conditions (Wu et al., 2017). Platelet microparticles also play a negative role in diabetes, similar to that in AKI and CKD, which is not only related to hypercoagulability, but also can promote glomerular endothelial cell damage (Zhang et al., 2018a; Yu et al., 2018).

### 3.4 The role of extracellular vesicles in renal tumor

In renal tumors, microRNA-210 and microRNA-1233 in circulation levels show statistically significant differences between healthy people and patients with kidney cancer (Zhang et al., 2018b). These differentially expressed microRNAs may not only serve as an indication for the outcome of tumors, but also have certain functions. Moreover, EVs secreted by cancer cells themselves can promote endothelial angiogenesis, which might be related to tumor metastasis and a potential target for detection and intervention (Grange et al., 2011; Gopal et al., 2016).

## 4 Urinary extracellular vesicles: Potential disease markers

The study of EVs in the kidney diseases commenced with the study of urinary EVs (Kanno et al., 1995). These EVs secreted in urine are often produced from the nephron or renal tubules. The components in these EVs, such as proteins or RNAs, can directly or indirectly reflect the physiology of the urinary system (Pisitkun et al., 2004). For example, markers such as podocin and podocalyxin for podocytes, Aquaporin-1 for the proximal tubule, CD9 and Type 2 Na-K-2Cl cotransporter for the thick ascending limb of Henle, and Aquaporin-2 for the collecting duct, all of these can be detected in urine EVs. The detectability of these markers of renal intrinsic components in urine EVs also demonstrates the great potential of urine exosomes (Pisitkun et al., 2004). Interestingly, recent studies have also shown that EVs in urine could also be originate from the circulation (Pazourkova et al., 2016). Due to the easy of sampling and availability of urine, EVs in urine have great potential for noninvasive diagnosis. However, due to individual differences, EVs in urine are difficult to quantify. At present, there are still many bottlenecks in this field.

Most of the EVs in urine originate from nephrons or renal tubules. They transport information from the original secretory cells to the downstream cells. Research based on urine EVs mainly focuses on the components, such as proteins or RNAs. These components can serve as diagnostic marker for kidney disease and have become a new noninvasive diagnostic tool or therapeutic target (Huebner et al., 2015; Zhang et al., 2016a; De Palma et al., 2016; Lv et al., 2019b). For example, microRNA 29c and CD2AP mRNA in urine EVs are related to kidney fibrosis; microRNA-451–5p and WT-1 mRNA are related to diabetes; microRNA-145, microRNA-141, microRNA-196a-5p, microRNA-501–3p, and CDH3 mRNA are related to prostate cancer; microRNA-204–5p is related to kidney cancer; and microRNA-21–5p is related to epithelial cancer (Lv et al., 2013; Lv et al., 2014; Filella and Foj, 2016; Mohan et al., 2016; Royo et al., 2016; Matsuzaki et al., 2017; Rodríguez et al., 2017; Xu et al., 2017; Abe et al., 2018; Kurahashi et al., 2019). Differences in exosome RNA or protein have been detected in urinary EVs of patients with bladder cancer, hypertension, and urinary tract infection (Lee et al., 2018; Perez-Hernandez et al., 2018; Mizutani et al., 2019). These differential expressions were found at early stage or advanced stage which were suitable for early diagnosis or progress detection, respectively. However, its specific application still needs furthers research due to factors such as individual variability. Although clinical application is difficult at present, these potential markers are milestones for subsequent research (*Table 1*).

**TABLE 1:** MiRNAs associated with kidney diseases in urine EVs.

Diseases	miRNAs in urine EVs	Model	Up or down	References
Diabetic Nephropathy	miR-145	Human	Up	Barutta et al. (2013)
	miR-451–5P	Rats	Up	Mohan et al. (2016)
Renal fibrosis	miR-29c	Human	Down	(Lv et al., 2013), (Solé et al., 2015)
Autoimmune glomerulonephritis	miR-26a	Human	Down	Ichii et al. (2014)
Urothelial carcinoma	miR-21–5p	Human	Up	Matsuzaki et al. (2017)
Bladder cancer	miR-375, miR-146a	Human	Down	Andreu et al. (2017)
Prostate cancer	miR-141	Human	Up	Mizutani et al. (2019)
	miR-196a-5p, miR-501–3p	Human	Down	Rodríguez et al. (2017)
	miR-21, miR-204, miR-375	Human	Up	Koppers-Lalic et al. (2016)
	miR-145	Human	Up	Xu et al. (2017)

Urine EVs contain proteins such as membrane proteins and transport proteins, which are related to kidney function. Aquaporins, particularly aquaporin 2, are critical proteins for kidneys to process water. The urine EVs containing aquaporin-2 can maintain a certain permeability, and the changes of these proteins can reflect the changes in kidney function (Sonoda et al., 2009; Abdeen et al., 2014; Miyazawa et al., 2018). In AKI caused by I/R, the secretion of aquaporin-1 and aquaporin-2 in urine exosomes is reduced, and the secretion of exosome-related markers such as Alix and TSG101 is increased at the later stage, suggesting that urinary exosome secretion increases during fibrosis (Asvapromtada et al., 2018). In an AKI model caused by cisplatin and in patients with polycystic kidney disease, the secretion of aquaporin-2 in the urine EVs decreases; detecting changes in these membrane proteins in the urinary EVs can help the diagnosis and evaluation (Pocsfalvi et al., 2015; Sonoda et al., 2019).

Acute T cell mediated rejection and acute antibody mediated rejection are very common in renal transplantation rejection, and lack of specific detection indicators (Cornell et al., 2008). Recent studies have shown that there are differences in plasma exosome RNA in acute antibody rejection, especially CCL4, which can be used as a predictive and diagnostic indicator of acute antibody mediated rejection (Zhang et al., 2017). Moreover, in acute T cell-mediated rejection, a urine exosome CD3 detection kit has been developed with high efficacy and clinical translation prospects (Park et al., 2017).

In addition to encapsulating some distinct proteins or small RNAs, urine EVs can also carry some specific molecules of the original cells, which can help in assessing the status of the original cells, and further reflect disease evaluation. Podocyte-specific EVs have been detected in urine EVs (Prunotto et al., 2013). These EVs contain podocyte-specific markers, such as nephrin, podocin, and WT-1, the urinary levels of which can reflect podocyte injury (Abe et al., 2018; Zhang et al., 2019). These podocyte-specific EVs can be sorted in urinary EVs for further study. Diabetic nephropathy is accompanied by damage to the structure and function of podocytes. Levels of podocyte-specific EVs in urine was increased in patient with diabetic, suggesting their role as a potential marker of diabetic nephropathy (Wu et al., 2018). Urinary podocyte-derived EVs are increase in patients with renal vascular hypertension (Kwon et al., 2017). These increased EVs may reflect subsequent podocyte damage after renal injury. In patients with preeclampsia, the proportion of podocin-positive and nephrin-positive EVs in urine has also increased (Gilani et al., 2017).

Moreover, some studies also focus on the unique modifications of urinary EVs and the differences in specialized detection methods. For example, the glycosylation modification of urinary EVs is different in various diseases and could be used as potential disease markers (Gerlach et al., 2013). However, the current problem is that Tamm Horsfall protein is also a highly glycosylated protein, which easily co-precipitates and is difficult to distinguish in research (Rosenfeld et al., 2007). Moreover, changes in the abundance of V-ATPase subunits in urine exosomes can reflect the kidney’s response to acid-base load; similarly, there are differences in Raman spectroscopy of urine EVs in patients with diabetic (Pathare et al., 2018; Roman et al., 2019).

The mechanism through which EVs meditate the exchange of information between cells is still questionable. A recent guess called genetic transfer was relatively new. The exosomes produced by kidney stem cells can release into the urine. On the one hand, it can predict the characteristics of the original cells, and on the other hand, it can reveal the status of the kidney (Bruno and Bussolati, 2013). CD133 is a renal stem cells marker and is highly expressed in the urethral endothelium of healthy people. CD133 level are decreased in urine EVs during acute and chronic glomerular injury, and in patients with vascular injury after transplantation. This decrease may be due to the fact that these abnormalities are accompanied by certain dysfunction of stem cells, and CD133 can also be used as a means of detecting EVs in urine (Dimuccio et al., 2014; Dimuccio et al., 2020).

At present, most of the EVs in urine are concentrated on biomarkers, and the functions of a few have been studied. Urine had been considered as a waste material of human metabolism, and it is routinely assumed that its EVs might not have practical functions. However, current research has shown the biological activity of urine exosomes. Part of the small RNA wrapped in urine exosomes can even act as a paracrine regulator of renal tubular transporters (Gracia et al., 2017). Klotho is mainly produced in the kidney and is related to kidney transporters and ion channels (Kim et al., 2017; Kuro-O, 2019). Research has confirmed that Klotho in urine EVs can promote the recovery of AKI (Grange et al., 2020).

## 5 Stem cell extracellular vesicles: Therapeutic applications

Stem cell and progenitor cell therapy has made corresponding progress in many fields, including kidney diseases (Cantaluppi et al., 2013; Wong et al., 2015). Mesenchymal stem cells and adipose stem cells promote the repair of AKI or CKD; however, the specific mechanism is still controversial (Kuppe and Kramann, 2016; Wankhade et al., 2016). There are two possible mechanisms for stem cells to participate in kidney repair. One is that stem cells directly differentiate to replace damaged kidney cells, and the other is that stem cells produce some cytokines and other special components to participate in kidney repair (Rossol-Allison and Ward, 2015). In particular, the second mechanism has aroused great interest in recent years. Stem cells produce EVs containing some cytokines produced by stem cells in different conditions and play function as a paracrine regulator to other tissues (Nargesi et al., 2017).

Current research confirms that the repair function of renal progenitors cells is mainly achieved by the paracrine functions of EVs. In AKI caused by I/R, EVs derived from stem cells can protect the kidney injury by inhibiting oxidative stress, reducing the numbers of NK cells, promoting angiogenesis through HIF-1α, and enhancing renal mitochondrial function (Zhang et al., 2014; Zou et al., 2016a; Zou et al., 2016b; Cao et al., 2020). These functions may be attribute to some special components of EVs in stem cells. These components may be proteins, nucleic acids, and some small RNAs. Mesenchymal stem cells derived from human pluripotent stem cells can inhibit necrosis by releasing specific proteins and activating the transcription of sphingosine kinase 1, thereby protecting kidney injury in I/R AKI (Yuan et al., 2017). Bone marrow mesenchymal stem cells produce miR-199a-5p-loaded exosomes to inhibit endoplasmic reticulum stress, thereby protecting the kidney from I/R AKI (Wang et al., 2019a). EVs derived from bone marrow stromal cells protect against AKI by enhancing NRF2/ARE activated antioxidants (Zhang et al., 2016b). Exosomes released from the human umbilical cord mesenchymal stem cells play a protective role in cisplatin-induced AKI by activating autophagy, ameliorating renal oxidative stress and apoptosis, and repairing cisplatin-induced renal tubules epithelial cell damage in rats (Zhou et al., 2013; Wang et al., 2017).

To overcome fibrosis, the EVs of stem cells have also demonstrated their protective functions. The EVs derived from stem cells possess anti-apoptotic activity and protect of renal tubular cells, which can inhibit the increase of mesangial substrate in diabetic mice and reverse the fibrosis process (Nagaishi et al., 2016; Collino et al., 2017; Grange et al., 2019). There are also studies using engineered stem cells, overexpressing of microRNA-let7c to reduce renal fibrosis. To address the issue of short half-life of EVs, studies have demonstrated that the loading of EVs with gel enhances the function of stem cell EVs (Wang et al., 2016; Zhou et al., 2019; Zhao et al., 2020). This “carrier” model is a promising strategy for the clinical application of stem cell therapy. In terms of renal tumors, EVs of human liver stem cells can inhibit tumor angiogenesis and tumor growth (Lopatina et al., 2019; Brossa et al., 2020). Renal clear cell carcinoma is the most common kidney tumor, and some patients manifested metastases at diagnosis (Barata and Rini, 2017). Exosomes isolated from tumor stem cells of patients with clear cell carcinoma transport miR-19b-3p, promote cancer cell proliferation, and epithelial mesenchymal transformation. In this process, tumor stem cell exosomes had been enriched in the lungs with CD103. After knocking out CD103 *in vitro*, the concentration of cancer cells in the lungs is greatly reduced. Therefore, CD103 may serve as one of the diagnostic indicators for early metastasis of renal clear cell carcinoma (Wang et al., 2019b).

## 6 extracellular vesicles as a therapeutic vector in kidney diseases

Number of studies have proved that particles with a size of about 100 nm, such as exosomes, can be metabolized by the kidney after passing through the blood, which provides a platform for exosome-mediated targeted therapy (Ma et al., 2020; Paluszkiewicz et al., 2021). EVs have good biocompatibility and are natural carriers. Nanocarriers formed by cell-derived membranes can inherit functions such as immune evasion, long circulation, and recognition ability of source cells (Li et al., 2021). Therefore, among them, EVs have been widely used as “natural treasures” in recent years (Vader et al., 2016). Methods for drug loading in exosomes involve direct combination, such as mixing the drug directly with the extracted exosomes, and indirect combination mainly including combining the drug with EVs through techniques such as electroporation and microfluidics (Elsharkasy et al., 2020). Cell membrane-coated carriers are nanocarriers prepared by coating synthetic nanoparticles swith a layer of natural cell membrane, which possess functions of, both, synthetic nanoparticles, and those of the source cells (Dash et al., 2020).

With regard to the kidneys, current studies have found that microvesicles produced by macrophages and stimulated by dexamethasone have improved effects on renal inflammation and fibrosis (Tang et al., 2019). This is a method of directly utilizing EVs drug loading, that is, to extract EVs after direct interaction between drugs and secretory cells and then intervene in other target cells. The EVs produced by the overexpression of IL-10 in macrophages have a good therapeutic effect on I/R AKI (Tang et al., 2020). More interestingly, the same team, who constructed targeting kim-1 peptides and therapeutic siRNAs into erythrocyte-derived EVs, found it useful in renal I/R injury and unilateral ureteral obstruction (Tang et al., 2021). Moreover, by “loading” the exosomes, the exosomes of IL-12-anchored kidney cancer cells have tumor rejection antigens, which have enhanced immunogenicity and anti-tumor effects (Zhang et al., 2010). Thus, the therapeutic effect of exosomes in conditions elated to the kidney has good application prospects.

## 7 Perspective and conclusion

EVs have been a research hotspot in recent years, with increasing progress in kidney diseases; However, their application is still relatively new research approach. EVs are important mediators of cell-to-cell information transmission in kidney diseases. For example, renal tubular cells promote the activation of M1 macrophages and the occurrence of interstitial inflammation in the condition of albumin stimulation, while macrophages stimulated by lipopolysaccharides also promote renal inflammation (Singhto et al., 2018; Lv et al., 2020). The exosomes-driven communication between cells is mainly achieved through the transmission of proteins and microRNA. Current studies suggest that exosomes are important carriers of microRNA transmission, but whether the function of exosomes can be realized by a single exosome in terms of information transmission is still unclear (Kalluri and LeBleu, 2020). Exosomes are initially secreted from plasma membrane invagination through early endosomes, late endosomes, and MVBs. Initial studies have confirmed that, in addition to endosome components and cytosolic special proteins, a few exosomes may also contain some organelle components, such as Golgi apparatus and nuclear components (Andreu and Yáñez-Mó, 2014). It has also been suggested that the exosome is not just a collection of cell debris, but rather a subcellular organelle. MicroRNA is an important functional component of EVs, which can regulate RNA and protein levels in recipient cells. These functional components wrapped in exosomes are efficiently protected from degradation. This further demonstrates the importance of EVs in intercellular communication and transfer of active components. The sorting mechanism of exosomes is very complex, which is mainly related to endosomal sorting complex required for transportation at present, but some studies have confirmed that exosomes can also complete sorting to form MVB through the ceramide pathway (Hanson and Cashikar, 2012). However, the mechanism of selectively sorting mrcroRNAs and proteins into exosomes is still unclear.

Exosomes have initially shown great potential in clinical diagnosis and treatment. The composition of urine exosomes varies among kidney diseases. These differential components, such microRNAs or proteins, may serve as important diagnostic tools in the future. For example, WT-1 in urine exosomes can diagnose early-stage diabetes (Kamińska et al., 2016). In addition, considerable research has recently focused on the purification of exosomes, such as immunoadsorption, and microfluidic and other methods to achieve trace extraction, which is an important step for urine exosomes to be used as a means of diagnosis and treatment (Gurunathan et al., 2019).

In addition, plasma EVs in different kidney diseases are also demand further study. Recent studies have also demonstrated that plasma EVs from patients with diabetic are enriched in microRNA4449 and can induce inflammation (Gao et al., 2021). Plasma EVs have a complex composition and are more difficult to study than are urine EVs, and there are many confounding factors. The study of plasma EVs in kidney diseases is also of great significance in clinical diagnosis and treatment.

Although considerable progress has been made in the study of EVs in kidney diseases, challenges in clinical translation still exist. Major issues focus on challenges in the purification and quantification of exosomes. Different extraction methods considerably influence on the purity. These bottlenecks affect the progress of current research. However, these difficulties will be gradually overcome in further research, and the role of EVs will be intelligible and easily applied in clinical practice.
